# Randomized Controlled Trial of Polyethylene Glycol versus Oral Sodium Phosphate for Bowel Preparation in Unsedated Colonoscopy

**DOI:** 10.1155/2020/6457079

**Published:** 2020-08-24

**Authors:** Dong Yang, Ke Tao, Geng Chen, Luping Zhang, Qingying He, Hong Xu

**Affiliations:** Department of Gastroenterology, The First Hospital of Jilin University, No. 1 Xinmin Street, Changchun, China 130021

## Abstract

**Aim:**

To identify the most effective laxative for bowel preparation in unsedated colonoscopy.

**Methods:**

Between April 2019 and April 2020, a total of 586 outpatients scheduled for unsedated colonoscopy at the First Hospital of Jilin University (Changchun, China) were randomized into one of two groups, namely, the polyethylene glycol (PEG) group or the oral sodium phosphate solution (OSP) group. The cleaning efficiency and other relevant clinical parameters were compared between the two groups.

**Results:**

Each group consisted of 293 patients. There were no significant differences in gender, body mass index, and history of abdominal surgery between the two groups. There were more cases of laxative intolerance in the PEG group than in the OSP group (7.5% vs. 0.7%, *P* < 0.05). After tube insertion, we found that the cleaning efficiency of OSP was better than that of PEG (*P* < 0.05). After cleaning, there was no significant difference in bowel cleanliness between the two groups (*P* > 0.05). The colonoscopic insertion time of the PEG group was significantly shorter than that of the OSP group (10.0 vs. 12.0 min, *P* = 0.002), and colonoscopic insertion was more difficult in the OSP group than in the PEG group (*P* = 0.036). The VAS score of the PEG group patients was significantly lower than that of OSP group patients (4.0 ± 1.3 vs. 5.2 ± 1.7, *P* ≤ 0.001). There were no significant differences in the cecal intubation rate and the detection rate of polyps and ulcers/erosion between the two groups.

**Conclusion:**

The cleaning efficiency and tolerability of OSP were preferable to those of PEG, but there was no significant difference in bowel cleanliness after washing the colon and suctioning the fluid. Compared with patients of the OSP group, those of the PEG group required a shorter colonoscopic insertion time and reported a more comfortable experience. Therefore, for cases that are tolerant of PEG, PEG is a better choice for unsedated colonoscopy.

## 1. Background

Adequate bowel preparation is the premise of successful colonoscopy, and inadequate bowel preparation has a detrimental effect on the procedure [1–3]. Although colonoscopy with sedation has been widely applied, some patients still choose unsedated colonoscopy [4, 5]. Because of the characteristics of unsedated colonoscopy, clinicians and medical staff need to be mindful of laxative tolerability and pain during colonoscopy [6] as well as the outcomes of the procedure. Interestingly, these three parameters are directly related to the laxative used. In clinical practice, however, different physicians follow different regimens. Although polyethylene glycol (PEG) has been recommended for general use, whereas oral sodium phosphate (OSP) has not been, the quality of evidence in guidelines is still low [7]. Furthermore, the results of different studies are controversial [8–10]; therefore, further investigations are needed. In our department, we use both PEG and OSP for bowel preparation in patients without contraindications. Therefore, this study compared the efficacy and tolerability of two laxatives to determine which one is more suitable for bowel preparation in unsedated colonoscopy.

## 2. Materials and Methods

### 2.1. Study Particulars

**Figure 1:**
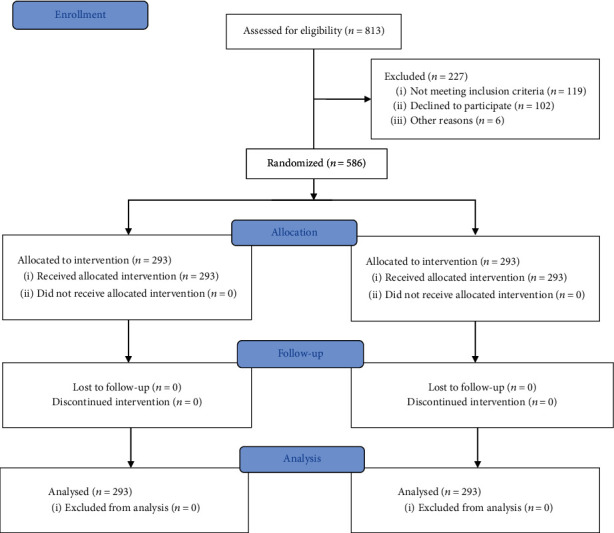
Flow chart detailing the conduct of the study.

This is a randomized controlled trial, which was evaluated and approved by the ethics committee at our institution, and registered in clinicaltrials.gov under number NCT03817788. Ethical approval for this study (19K020-001) was provided by the ethics committee of the First Hospital of Jilin University, Changchun, China (Chairperson Professor J. Jiang), on 26 April 2019. Between April 2019 and April 2020, a total of 586 outpatients scheduled for unsedated colonoscopy at the First Hospital of Jilin University (Changchun, China), who met the inclusion criteria and provided informed consent, were randomized into one of two groups, namely, the PEG group or the OSP group. Randomization was carried out according to the randomization table, before ingesting the laxative. Investigators assessing outcomes and analyzing data were blinded to the clinical characteristics of the patients. (Figure 1).

The inclusion criteria were as follows: (1) patients aged between 18 and 60 years, (2) patients whose cardiopulmonary function could tolerate unsedated colonoscopy, and (3) those with good general health. The exclusion criteria were as follows: (1) patients with an ASA score ≥ 3; (2) patients with a history of colorectal resection, except appendectomy; (3) patients with an obstruction, an incomplete obstruction, or a lower digestive tract hemorrhage; (4) those with a history of kidney disease; (5) patients unable to strictly adhere to the guidelines of fluid intake; and (6) patients with a history of preexisting electrolyte disturbances, (7) inflammatory bowel disease or delayed bowel transit, (8) parathyroidectomy, (9) heart disease, (10) diabetes, (11) pregnancy, and (11) antidepressant and/or opioid use.

All patients received instructions on a unified diet, and a low-residue diet was recommended before colonoscopy. In this study, the effects of one type of polyethylene glycol solution and one type of oral sodium phosphate solution were assessed. The day before the colonoscopy, the patients of the PEG group consumed 750 mL of sulfate-free polyethylene glycol electrolyte solution (Freecol, Staidson Biopharmaceuticals Inc., Beijing, China) 2 hours after dinner, and 4–6 hours before the colonoscopy, the patients of this group consumed another 1500 mL of solution. On the other hand, the day before the colonoscopy, the patients of the OSP group consumed 750 mL of fluid containing 45 mL of sodium phosphate solution (Danfang, Jewelland Pharm Inc., Sichuan, China) 2 hours after dinner, and the same volume was ingested 4–6 hours before the colonoscopy. To prepare the bowels for the procedure, the patients were strongly advised to increase their fluid intake. Subsequently, the patients were given a 200 mL solution containing 400 mg of simethicone, an antifoaming agent.

All patients were examined by three experienced endoscopists with more than 5 years of experience and more than 1000 performed colonoscopies per year, and the relevant indexes were recorded. Bowel cleanliness was assessed during tube insertion using a four-point scale as follows: P for poor, F for fair, G for good, and E for excellent [11]. The lumen was thoroughly washed, and the fluid was suctioned. Cases requiring additional water to lubricate the lumen, so as to reduce the difficulty of colonoscopic insertion and to alleviate the pain, were recorded. The difficulty of colonoscopic insertion was reported using a three-point continuous scale as follows: E for easy, M for moderately difficult, and D for difficult. During tube withdrawal, the Boston Bowel Preparation Scale (BBPS) [12] was used to reassess the quality of the bowel preparation. The withdrawal time should be ≥6 minutes. The detection of polyps of any size and ulcers/erosion should be recorded, and biopsy specimens should be obtained. The degree of pain experienced during the entire procedure was communicated by the patient using a visual analogue scale (VAS) as follows: 0 for no pain and 10 for worst imaginable pain [13].

### 2.2. Statistical Analysis

This study is a randomized controlled trial designed for noninferiority to explore whether the effect of OSP on bowel cleanliness is not inferior to that of PEG. Based on the results of our team's previous small sample observational study, 75.2% of the patients with PEG had a bowel cleanliness grade of good or excellent. Assuming that the cleaning effect of OSP is not inferior to that of PEG, the noninferiority boundary value is 10%. If *α* = 0.025 (one side), 1-*β* = 0.8, and the two groups are equal, the sample size of the experimental group and the control group is *N*1 = *N*2 = 293 cases. All statistical analyses were performed using SPSS software (ver. 20.0 for Windows). Comparisons between the groups were performed by applying the chi-squared (*χ*^2^) test (for categorical variables), Mann–Whitney *U* test and Kruskal–Wallis test (for non-normally distributed continuous variables), and *t*-test (for normally distributed continuous variables). A two-tailed *P* value < 0.05 was considered statistically significant.

## 3. Results

A total of 586 cases were included in this study, and each group was comprised of 293 patients. The baseline data are shown in Table 1. There were no significant differences in gender, body mass index (BMI), and history of abdominal surgery (*P* > 0.05). However, the patients of the PEG group were older than those of the OSP group (46.4 ± 9.8 vs. 43.3 ± 10.5 years, *P* ≤ 0.001). There were no cases of kidney injury requiring medical intervention or other complications requiring surgical intervention during the entire study. However, there were cases of laxative intolerance in both groups (22 patients in the PEG group and two patients in the OSP group), with symptoms of abdominal distention, nausea, vomiting, and an inability to take all laxatives, and the results were significantly different (7.5% vs. 0.7%, *P* ≤ 0.001) (Table 1).

Values are presented as numerals and/or percentages, with the latter shown in parentheses.

During colonoscopic insertion, we found that 92.8% of patients in the PEG group had a bowel cleanliness grade of good or excellent, which was significantly lower than that of patients in the OSP group (96.6%) (*P* = 0.001). The cecal intubation rate was higher in the PEG group than in the OSP group (99.7% vs. 98.3%, *P* = 0.218), although the results were not significantly different. In addition, we found that colonoscopic insertion was moderately difficult or difficult in 23.2% of PEG group patients compared to 32.8% of OSP group patients, and the results were significantly different (*P* = 0.036). The rate of patients in the PEG group requiring additional water to lubricate the lumen, so as to reduce the difficulty of tube insertion and alleviate the pain, was significantly less than that in the OSP group (19.1% vs. 72.7%, *P* ≤ 0.001). The colonoscopic insertion time was significantly shorter in PEG group patients than in OSP group patients (10.0 vs. 12.0 min, *P* = 0.002), and the VAS score of PEG group patients was significantly lower than that of OSP group patients (4.0 ± 1.3 vs. 5.2 ± 1.7, *P* ≤ 0.001). (Table 2).

Values are presented as numerals and/or percentages, with the latter shown in parentheses.

After washing and suctioning the bowel, the efficacy of the laxatives was assessed by the BBPS. There was no significant difference between the two groups (8.5 ± 1.0 vs. 8.5 ± 0.9, *P* = 0.567), indicating that both laxatives were equally effective in cleaning the transverse and right colon. However, the cleanliness score of the left colon was significantly higher in patients of the PEG group than in patients of the OSP group (3.0 ± 0.2 vs. 2.9 ± 0.4, *P* = 0.001). There were no significant differences in the detection rates of polyps and ulcers/erosion between the two groups (*P* > 0.05) (Table 3).

Values are presented as numerals and/or percentages, with the latter shown in parentheses.

## 4. Discussion

There are few studies in the literature comparing the efficacies of PEG and OSP in cleaning the bowel for colonoscopy [8–10, 14]. This study focused on unsedated colonoscopy [6]. Furthermore, there is no reported case of severe kidney injury or another related complication caused by the use of OSP, which may be related to the colonoscopy protocol followed by our center, whereby patients consume at least 1700 mL of fluid to protect the function of the kidneys [15].

Our results revealed that there were significantly more laxative intolerant patients in the PEG group than in the OSP group. These results can be explained by the difference in fluid intake (2450 mL for the PEG group vs. 1700 mL for the OSP group) and the unappealing taste of PEG, which may have caused nausea and bloating in patients [16]. Therefore, the tolerance of patients to OSP was better.

We found that the cleaning efficiency of OSP was better than that of PEG, which is inconsistent with the results of previous studies [9, 10]. After washing and suctioning according to BBPS standards, we found no significant difference in the cleaning efficiency between the two groups. Furthermore, the cleanliness score of the left colon in PEG group patients was significantly higher than that in OSP group patients, which may be explained by the fact that the BBPS evaluates not only the cleaning efficiency of the laxative but also the effectiveness of the subsequent steps of washing and suctioning. As PEG is taken with large volumes of fluid, it passes through the bowel without the net movement of fluid and electrolytes across the colonic membrane, so what remains in the lumen is a mixture of solids and opaque liquid. Although the cleaning efficiency of PEG was poorer than that of OSP, the washing and suctioning steps were effortless. By contrast, OSP is hyperosmotic [16], drawing surrounding fluid into the lumen to stimulate a bowel movement; therefore, most residue was solid and difficult to remove. Therefore, there was no significant difference in the cleaning efficiency between the two groups after suctioning the opaque liquid. Previous studies have reported that the quality of the bowel preparation is correlated with other quality measures such as the cecal intubation rate and adenoma detection rate [17, 18]. In this study, there were no significant differences in the cecal intubation rate and the detection rate of polyps and ulcers/erosion between the groups, consistent with the results of Chaussade et al. [8]

We found that colonoscopic insertion was significantly more difficult in patients of the OSP group than in patients of the PEG group, and the insertion time was significantly shorter in PEG group patients than in OSP group patients. These findings can be explained by the fact that PEG is an inert, nonabsorbable, and isosmotic agent capable of drawing the orally consumed fluids into the colon and effectively lubricating the colorectal mucosa, thereby facilitating colonoscopic insertion. By contrast, OSP is a hyperosmotic agent capable of drawing surrounding fluids into the lumen, and as a result, the colorectal mucosa is dehydrated and sticky, thereby complicating colonoscopic insertion. The additional water here was used not for better bowel cleanliness, but for lubricating the colorectal mucosa, and the rate of patients in the PEG group requiring additional water to lubricate the lumen was significantly lower than that in the OSP group (19.1% vs. 72.7%, *P* ≤ 0.001). This interesting finding is consistent with the study by Lee et al. [19] All patients underwent unsedated colonoscopy, which allowed us to obtain real-time data on the subjective feeling of pain. The results showed that the VAS of PEG group patients was significantly lower than that of OSP group patients, consistent with results on the difficulty of colonoscopic insertion and colonoscopic insertion time. Taken collectively, these results demonstrate that PEG is a better choice for inexperienced endoscopists.

There were several limitations in this study. Presently, it is still uncertain whether OSP can damage kidney function [20, 21]. Here, we examined the efficiency and tolerability of two laxatives in patients, and renal function was not routinely assessed because of limited resources. We also wished to avoid all causes of unnecessary distress on patients. Although no kidney injury or related complication was observed, we could not assess the potential risk of minor kidney injury caused by OSP or PEG. Therefore, further studies are needed to investigate this postulate.

In conclusion, compared to PEG, the cleaning efficiency and tolerability of OSP were preferable, but there was no significant difference in bowel cleanliness after washing and suctioning between the two laxatives. Compared with patients of the OSP group, those of the PEG group required a shorter colonoscopic insertion time and reported a more comfortable experience. Therefore, for cases that are tolerant of PEG, PEG is a better choice for unsedated colonoscopy.

## Figures and Tables

**Table 1:** Baseline and clinical data of the patients.

Variable	PEG group	OSP group	*P* value
Age (yr)	46.4 ± 9.8	43.3 ± 10.5	≤0.001
Gender			0.619
Male	162	156	
Female	131	137	
Body mass index	23.8 ± 3.5	23.9 ± 3.7	0.666
History of abdominal surgery			0.858
Yes	90	92	
No	203	201	
Tolerance			≤0.001
Yes	271 (92.5)	291 (99.3)	
No	22 (7.5)	2 (0.7)	

**Table 2:** Clinical data relating to colonoscopy tube insertion.

	PEG group	OSP group	*P* value
Bowel preparation			0.001
Poor	1 (0.3)	0	
Fair	20 (6.8)	10 (3.4)	
Good	115 (39.2)	79 (27.0)	
Excellent	157 (53.6)	204 (69.6)	
Cecal intubation			0.218
Yes	292 (99.7)	288 (98.3)	
No	1 (0.3)	5 (1.7)	
Cases requiring additional water to lubricate the lumen	56 (19.1)	213 (72.7)	≤0.001
Difficulty of colonoscopic insertion			0.036
Easy	225 (76.8)	197 (67.2)	
Moderately difficult	58 (19.8)	81 (27.6)	
Difficult	10 (3.4)	15 (5.1)	
Insertion time (min)	10.0 (8.0-14.0)	12.0 (9.0-15.0)	0.002
Visual analogue scale	4.0 ± 1.3	5.2 ± 1.7	≤0.001

**Table 3:** Clinical data relating to colonoscopy tube withdrawal.

	PEG group	OSP group	*P* value
Boston Bowel Preparation Scale	8.5 ± 1.0	8.5 ± 0.9	0.567
Left colon	3.0 ± 0.2	2.9 ± 0.4	0.001
Transverse colon	2.9 ± 0.4	2.9 ± 0.3	0.562
Right colon	2.7 ± 0.6	2.7 ± 0.5	0.389
Detection of polyps			0.534
Yes	96 (32.8)	89 (30.4)	
No	197 (67.2)	204 (69.6)	
Detection of ulcers/erosion			0.716
Yes	15 (5.1)	17 (5.8)	
No	278 (94.9)	276 (94.2)	

## Data Availability

The principal investigator, Dong Yang, had full access to all of the data in the study and takes responsibility for the integrity of the data and the accuracy of the data analysis.
